# Current Strategies for the Production of Sustainable Biopolymer Composites

**DOI:** 10.3390/polym13172878

**Published:** 2021-08-27

**Authors:** Ehsan Bari, Asghar Sistani, Jeffrey J. Morrell, Antonio Pizzi, Mohammad Reza Akbari, Javier Ribera

**Affiliations:** 1 Department of Wood Sciences and Engineering, Technical Faculty of No. 2, Mazandaran Branch, Technical and Vocational University (TVU), Sari 4816831168, Iran; sistani.asqar@yahoo.com; 2 National Centre for Timber Durability and Design Life, University of the Sunshine Coast, Brisbane, QLD 4102, Australia; jmorrell@usc.edu.au; 3 LERMAB-ENSTIB, University of Lorraine, 27 rue Philippe Seguin, 88000 Epinal, France; antonio.pizzi@univ-lorraine.fr; 4 Department of Wood and Paper Sciences, Tarbiat Modares University, Jalal AleAhmad, Nasr, Tehran P.O. Box 14115-111, Iran; reza.akbari.q@gmail.com; 5 Laboratory for Cellulose & Wood Materials, Empa-Swiss Federal Laboratories for Materials Science and Technology, CH-9014 St. Gallen, Switzerland

**Keywords:** biocomposites, circular economy, biodegradability, environmentally friendly

## Abstract

Rapid global population growth has led to an exponential increase in the use of disposable materials with a short life span that accumulate in landfills. The use of non-biodegradable materials causes severe damage to the environment worldwide. Polymers derived from agricultural residues, wood, or other fiber crops are fully biodegradable, creating the potential to be part of a sustainable circular economy. Ideally, natural fibers, such as the extremely strong fibers from hemp, can be combined with matrix materials such as the core or hurd from hemp or kenaf to produce a completely renewable biomaterial. However, these materials cannot always meet all of the performance attributes required, necessitating the creation of blends of petroleum-based and renewable material-based composites. This article reviews composites made from natural and biodegradable polymers, as well as the challenges encountered in their production and use.

## 1. Introduction

The extensive industrialization of polyethylene during the 1950s resulted in the mass production of petrochemical-derived plastics (i.e., polyethylene, polypropylene, polystyrene, and polyvinyl chloride) that remain widely used. Due to the versatile nature of plastics, they are widely used in textiles, packaging, building and construction materials, transportation, electronics, and industrial machinery. The majority of monomers used to produce plastics, such as ethylene and propylene, are derived from fossil hydrocarbons, meaning that none of the aforementioned plastics are biodegradable. As a result, in 2015 over 6300 million tons of plastic waste were generated worldwide; approx. 9% recycled, 12% incinerated and 79% accumulated in landfills or natural environments [1]. Present production, consumption, and waste management trends will result in an estimated 12,000 million tons of plastic accumulating by 2050 [1]. Thus, without appropriate management strategies for end-of-life plastics, the major terrestrial and aquatic ecosystems on this planet could be severely contaminated.

For this reason, many countries have launched special programs to eliminate and/or recycle plastic materials from the biosphere and have implemented novel strategies for the transformation of plastic waste into useful products or energy [2,3,4]. There has been increasing interest in the effective development and production of inexpensive bioplastics with properties comparable to conventional plastic materials based on substances such as starch, cellulose, and polylactic acid (Table 1) [4,5,6,7].

While these materials have promising properties, they also face challenges to replace traditional plastic-based materials including brittleness, low thermal stability, and poor barrier properties. Continued research and development have resulted in new biomaterials such as beverage containers, starch-based cutlery, and packaging. However, novel biomaterials should resist degradation for most applications. Thus, these materials may have some of the same end-of-life issues as petroleum-based polymers. An important breakthrough in the evolution of bio-based polymers will be the development of fully biodegradable materials.

Biodegradable polymers such as PHA, PHB, PLA, and TPS generally contain ester, amide, or other easily degradable bonds in their backbones and can be grouped into two large groups based on their structure and synthesis [8]. Agro-polymers are derived from biomass and primarily depend upon the existing polymer linkages for their properties. Agro-polymers include polysaccharides, such as starches found in potatoes or wood, and proteins, such as animal-based whey or plant-derived gluten [9]. Many of these polymers, such as starches and casein, have a long tradition as natural adhesives [10]. Most of these materials are readily biodegradable and their use is often limited by their susceptibility to environmental conditions.

In this review, the source of composites made from natural and biodegradable polymers, as well as the challenges that are encountered in their production and use, are presented and discussed.

## 2. Agro-Polymers (Fibers)

Most agro-polymers are used in fiber form. Fibers are natural or synthetic substances that are significantly longer than their width or diameter. Fibers are used in the manufacture of many engineering materials and can be (i) short or discontinuous fibers with aspect ratios between 20 and 60 mm and (ii) long or continuous fibers with aspect ratios between 200 and 500 mm that can be converted into strands and woven into fabric.

Natural fibers can be categorized as cellulose/lignocellulose fibers/plant fibers, animal fibers, or mineral fibers. Lignocellulosic (LC) fibers are also known as plant fibers, natural fibers, or vegetable fibers and include bast (or stem or soft sclerenchyma) fibers, leaf or hard fibers, seed, fruit, wood, cereal straw, and other grass fibers. Fibers can be extracted from wood pulp, cotton, bark, nut shells, bagasse, corn cobs, bamboo, cereal straw, and vegetable products such as flax, jute, hemp, sisal, and ramie. Plant-based fibers are mainly composed of cellulose, hemicellulose, lignin, and pectin along with small quantities of extractives. The fiber composition varies depending on its origin [11,12] (Table 2). The most commonly used natural fibers are sisal, hemp, basalt, kenaf, flax, wood, and bamboo [13].

Sisal is one of the most widely used natural fibers and Brazil is one of the largest suppliers of this biomaterial. Sisal is used for automotive components, shipping, and civil construction. It has an application range from the fiber core of steel wire cables in elevators to simple agricultural twine [14,15]. Hemp is commonly grown in northern temperate climates and grows up to 1.2–4.5 m high and 2 cm in diameter [16]. The inner girth contains short fiber cells that can be used for composites, while the outer layer contains the bast fibers that are used in rope, textiles, garden mulch, an assortment of building materials, and animal bedding. Hemp has also been used for composite production [17,18]. Kenaf is in the genus *Hibiscus* and produces bast fibers that are mainly used for paper and rope production [19]. Plant fibers are stiff, strong, and tough, but can also be converted into fine woven fabrics. Both kenaf and hemp are completely biodegradable.

Sisal, hemp, and kenaf fibers were traditionally used to produce textiles, cords, ropes, storage bags, and even boats. These fibers are also combined with other materials to produce hybrid composites for automotive components, construction materials, packaging, furniture, textiles, mats, and paper pulp [20,21].

Bio-polyesters are produced by microorganisms or synthesized from natural or synthetic monomers. For instance, polylactic acid has been the subject of extensive research because it is easily produced from corn and other starchy grains. These biopolymers are potentially biodegradable but can be recalcitrant in some applications such as starch-based cutlery, which decomposes very slowly in composting operations compared to other components [22,23]. As a result, portions of the cutlery may still be present when all other materials have been successfully composted. The non-degraded compounds must be screened out, adding cost, or left to compost for a longer period in order to obtain complete degradation. However, the remainder of the compost also continues to decompose, reducing yield and decreasing efficiency. Thus, there is a clear necessity for the development of biopolymers with more predictable degradation pathways at the end of life.

## 3. Biocomposites

**Figure 1:**
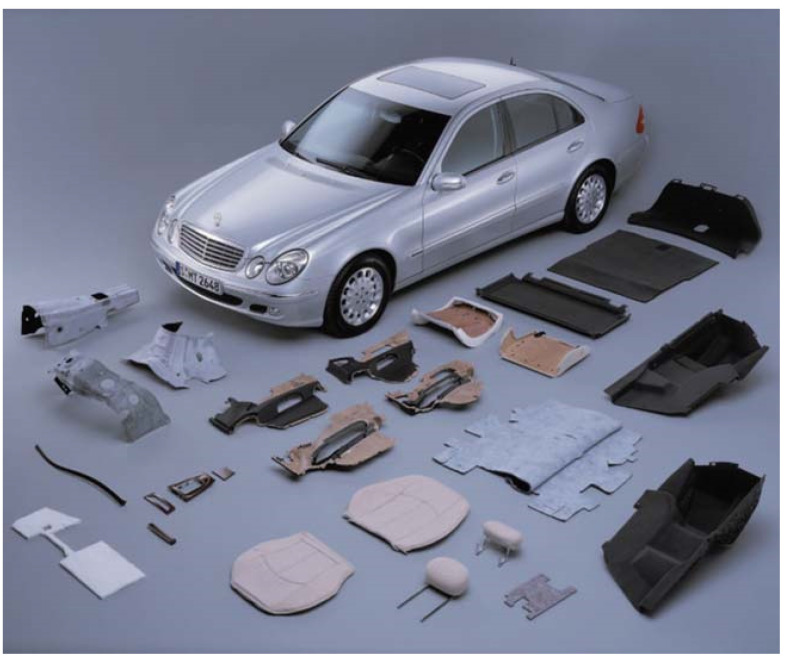
Examples of biocomposite applications in automobiles containing flax, hemp, sisal, and wool [26].

Biocomposites consist of one or more phases derived from biopolymers or renewable resources. Biocomposites mainly consist of natural fibers as reinforcements embedded within a biopolymer matrix [24]. This approach, in essence, mimics the natural fiber structure on a larger scale with the matrix surrounding fibers that contribute to composite stiffness. Biocomposites have been part of many traditional materials used by humans. For example, bows constructed from adhesive-bonded laminates of animal horns and tendons date back centuries. In recent years, there has been increasing interest in developing novel biocomposites that take advantage of the best properties provided by nature. Automobiles, packaging materials, and household products are major applications for natural-fiber reinforced biocomposites [25,26] (Figure 1).

Natural fiber-reinforced composites are used for both interior and exterior automobile parts because of their low weight and excellent isolation properties [27]. Sisal, palm, jute, and wood fibers have been explored to use in polyester, epoxy, and phenolic hybrid materials and have also been used for building materials such as laminates, door frames, door shutters, and partitions. A number of studies [28,29] indicate that laminates and panels using sisal, jute, and coir fibers in combination with polyester, phenolic, or polyurethane resins provide unique properties that are comparable to synthetic alternatives. Natural fiber-reinforced composites also represent a promising alternative for wood in construction materials [24] (Table 3).

## 4. Composites Made from Biopolymers

Biodegradable polymers constitute a loosely-defined family of polymers that can be metabolized by living organisms at the end of their service life. Commercially available biodegradable polymers include polycaprolactone (PCL), polylactic acid, polyhydroxyalkanoates, poly(ethylene glycol), and aliphatic polyesters, such as poly(butylene succinate) and poly(butylene succinate-co-butylene adipate) [31]. Biodegradable polymer fibers such as flax, hemp, and kenaf have all been explored as potential composite reinforcement materials to replace glass fibers because of their significant mechanical properties such as specific modulus and strength [32] (Table 4).

**Figure 2:**
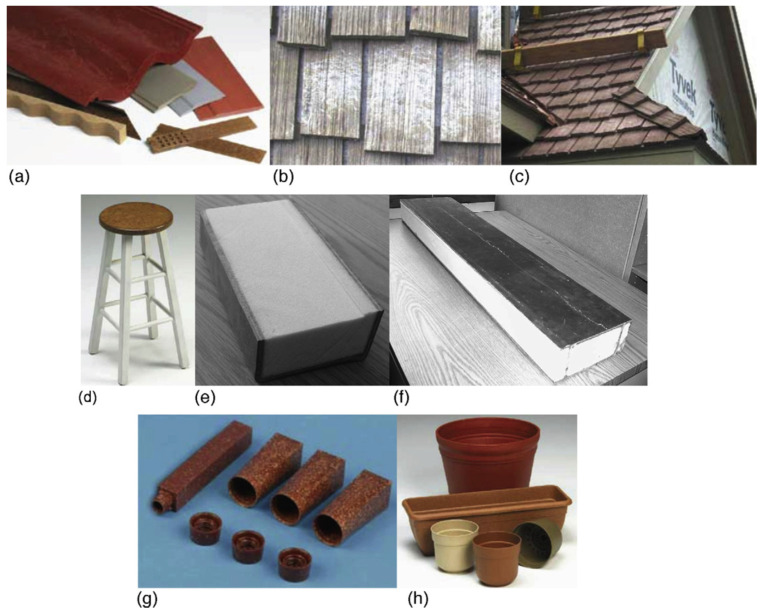
Examples of commercial biodegradable composites. (**a**–**c**) Building components [35]; (**d**) furniture [35]; (**e**) a chicken–soybean oil resin-based composite beam [36]; (**f**) a paper–soybean oil resin-based composite beam [37]; (**g**) cosmetic packing [35]; and (**h**) flower pots [35].

Biodegradable composites can be segregated into those that contain some level of synthetic polymers or resin that limits complete biodegradation and those containing reinforcement and matrix materials derived from renewable sources such as starch, proteins, polyvinyl alcohol (PVA), PLA in their native or modified forms that can be completely degraded by microorganisms [34]. A variety of commercial applications have been developed for these materials (Figure 2).

**Figure 3:**
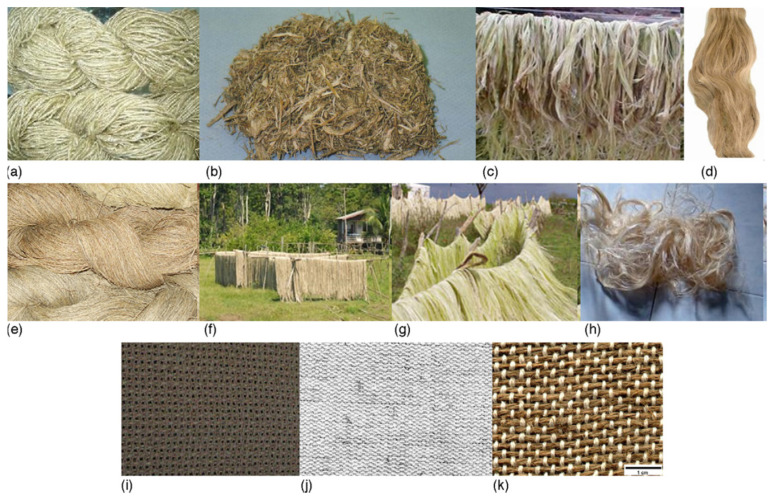
Examples of lignocellulosic reinforcement materials including. (**a**) banana stem fibers; (**b**) sugarcane bagasse; (**c**) curauá; (**d**) flax; (**e**) hemp; (**f**) jute; (**g**) sisal; and (**h**) kenaf. Typical reinforcement patterns used in hybrid LC-based biodegradable composite synthesis. (**i**) Jute fabric; (**j**) ramie–cotton fabric. (**k**) jute–cotton fabric. [40].

Although bio-based materials are available, abundant, low cost, and have unique properties, they do not provide all the desired properties and are relatively rapidly degraded. Moreover, they can be difficult to manufacture into the desired shapes and sizes. In addition, natural materials show considerable variations in their properties and it can be difficult to adapt existing equipment to process these materials. Similarly, bio-based materials have more limited mechanical properties than non-renewable sources. However, the diverse array of bio-based materials creates a range of possibilities for designing composites for specific applications or with specific material properties. Parameters such as filler concentration, geometric shape, and aspect ratio as well as the degree of interfacial adhesion between the filler and the matrix can all be tuned to produce a composite with optimal properties [38]. One approach to overcome the limitations of the natural materials is to combine them to utilize the unique properties of each component material [39]. In this approach, natural fibers (such as jute, hemp, sisal, oil palm, kenaf, and flax) are utilized as fillers or reinforcing material for polymer-based matrices [40], reducing waste disposal problems, and decreasing fossil fuel use (Figure 3).

Hybrid composites use more than one type of reinforcement and/or matrix and can include both inorganic and organic materials or combine synthetic polymers with natural fibers to enhance composite properties [41,42,43]. For example, jute has been used to produce a hybrid composite with oil palm, glass, polypropylene, sisal, and coir fibers. Ahmed and Vijayaragan [44] found good agreement between actual and predicted properties of hybrid laminates of woven jute and glass fabric in a polyester resin matrix for in-plane elastic properties under tension using the rule of hybrid mixtures and classical lamination theory models with a deviation up to about 20%. Jawaid et al. [33] showed that chemical resistance, void content, and tensile properties of tri-layer hybrid composites of oil palm empty fruit bunches and jute fibers could be varied by the placement of each material on the surface or core in an epoxy resin matrix. Gujjala et al. [45] investigated tensile, flexural, and interlaminar shear properties of hand-laid-up four plies and five types of hybrid laminates using woven jute and E-glass mat in an epoxy resin matrix. Maximum tensile and flexural strength were obtained with composites composed of glass/jute/glass (84 MPa) and jute/glass/jute/glass (162 MPa) sequences keeping the same volume fraction percentage (17.5%), while maximum interlaminar shear strength was observed for the composite prepared with glass fiber used as the extreme layers. Ramesh et al. [46] studied mechanical properties such as tensile strength, flexural strength, and impact strength of sisal–jute–glass fiber reinforced polyester matrix hybrid composites. Mohanty et al. [47] used a similar approach to produce composites with a range of mechanical properties (Table 5). They used chopped sisal and jute fibers of 30 mm and glass fiber layers to produce a five-layer hybrid composite in which glass fiber layers were fixed in the top, middle, or bottom of the specimens.

## 5. Challenges for Degradation of Sustainable Composites

The development of more eco-friendly composites with enhanced sustainability faces challenges for large-scale applications. Measuring the sustainability of plastic and reinforcement/fillers is a complex task affected by factors such as the origin of the feedstock, energy input during production, durability, health impacts, and end-of-life recycling or disposal [56]. Biomass supply chains are complex and encompass different types of biomass, harvesting and collecting strategies, transport and storage mechanisms, as well as processing methodologies. An important component of developing sustainable practices is to establish a unified protocol for the effective utilization of bioresources, including waste residues. For example, a more sustainable method for expanding purpose-grown biomass is to use marginal agricultural land. This approach would allow increased production without affecting food resources on more productive land. Durability is a critical test for any biocomposite replacing traditional synthetic composites. Biocomposites for automotive, construction, and other structural applications must deliver the required service life and long-term durability. The inclusion of bioplastic and recycled materials in sustainable composites also poses major scientific challenges because of the need to combine materials with different properties. Designing and engineering new classes of biocomposite materials that exhibit high tolerance against various external factors are essential. The classification of biodegradable and nonbiodegradable composites is also important from an application perspective. While many biocomposites are destined for applications where long service life under more extreme conditions will be essential, some materials are targeted for short life cycles. Creating materials that are easily recyclable or compostable becomes critical in these applications.

One challenge to recycling biocomposites is the co-location of composting facilities at the disposal site to avoid added transportation costs [47]. Biocomposites may contain one or more naturally derived components which can be part of the reinforcement phase or matrix phase or both in a composite system and may include: (i) biofiber-reinforced petro-based polymers (nondegradable), (ii) biofiber-reinforced biobased-polymers (biodegradable), and (iii) synthetic fiber (glass or carbon fiber)- reinforced biobased-polymers (nonbiodegradable) [57]. The ability to segregate these materials will be critical for creating effective recycling/reuse strategies.

Biofibers generally have minimal resistance to environmental degradation under the proper conditions, making them good candidates for reducing waste production [30]. Biodegradable polymeric materials include starch, chitosan, chitin, cellulose, lignin, polyla tic acid (PLA), poly(3-hydroxybutyrate (PHB), poly(3-hydroxybutirate-3-hydroxyvalerate) (PHBV), poly (butyrate adipate-co-terephthalate (PBAT), and polycaprolactone (PCL). It is important to note that some nonbiopolymers are also biodegradable while some bioplastics can be synthesized to reduce biodegradation. It is also important to understand the specific conditions and the timeframe under which a ‘biodegradable’ polymer actually decomposes. For example, most packaging materials marked as ‘biodegradable’ completely break down when composted in industrial units but have little opportunity to decompose under the anaerobic conditions present in most municipal solid waste facilities. Thus, establishing accurate degradation times can be challenging because of the wide array of possible conditions to which the material will be exposed. For example, it is desirable for plastic mulch film to completely photodegrade before the following crop cycle, to avoid soil burial of incompletely degraded plastic fragments [58].

In most other cases, however, the long-term durability of plant-based composites remains a concern since both the polymer matrix (bio-based) and biofiber components can be degraded under optimal conditions [59]. Exposure conditions also play an important role. For example, lignin is more susceptible to UV degradation, while hemicellulose is more prone to biological and moisture degradation [60].

## 6. Biological Degradation of Sustainable Composites

Many efforts have been made to design composites that are stable during their service life but susceptible to microbial attack under the proper conditions [61]. The addition of natural fillers to composites has been widely discussed in the literature because of their effects on the mechanical properties but they also influence composite degradability [62]. Cellulose-based additives are inherently hygroscopic and the inclusion of a sufficient percentage sharply increases the opportunity for some degradation to occur. However, natural composites may cause serious problems in construction materials because of this moisture behavior since it can lead to unacceptable deformation and, eventually, degradation [63]. The increasing proportions of hydrophobic polymers reduce this risk but also tend to increase cost, weight, and eventually ability to be composted.

Biodegradable plastics tend to undergo relatively rapid degradation over several months to several years (depending on the material and degradation conditions). These plastics are biodegradable according to the European Union Standards (EN 13432) [64] and can be organically recycled in compost. Biodegradable composites made of polylactic acid (PLA) are increasingly used in food packaging. However, manufacturers must find a balance between the mechanical and physical properties of these composites and their rapid degradation during composting. As a result, these materials are still under intensive exploration. Polyhydroxyalkanoates (PHAs) have a very important advantage for composite applications since their polar character results in better adhesion to lignocellulose fiber [65]. PHA composites with vegetable or grain fillers may find an application in horticulture as environmentally friendly low-cost crop containers intended to replace petroleum-based polymers. The main advantage of this solution is that the materials will degrade in the surroundings after use [66]. PHA composites could also be used for fresh fruit or vegetable packaging since the material would degrade simultaneously to the food residues during the composting process [67].

The biodegradation rate of composite materials depends on an array of factors including the monomers used, and the strength of polymer bonds, as well as the environmental conditions (e.g., temperature, moisture and pH of the soil, microbial population, and nutrient supply) to which the material is exposed [68]. Additionally, the surface area of the materials also influences the microbial access to the material and therefore, its compostability. For example, a rough surface with a high number of polar hydrophilic functional groups is much more prone to biodegradation than a smooth, hydrophobic, and inert one [69]. Natural fillers that are hydrophilic and more biodegradable increase the adhesion of microorganisms to the composite material and facilitates degradation. For example, a 40 µm thick Polybutylene succinate (PBS) polymers film, degrades at a rate of 50 % per 1 month in garden soil [70]. Although not as widely considered during the development process, improving the understanding of the susceptibility of each composite component to wetting and degradation (either physical or biological) can help to design more sustainable materials with higher performance but the ability to rapidly decompose into the surroundings at the end of their service life. The large surface area at the matrix–filler interface represents a weak zone that can limit use in some applications since the interface can act as an access point for degrading agents, but it also acts as a pathway for their eventual destruction [71]. The characteristics of the lignocellulose fibers can also affect degradation rates, with amorphous regions more likely to sorb moisture than crystalline regions and therefore begin to degrade [72]. Wetting effects can be very localized with small increases in moisture content creating large mechanical forces that disrupt the polymer matrix, allowing further moisture intrusion and eventual microbial attack [73].

Wood-plastic composites (WPC) usually contain 30–55% polymer matrix (PP, PE, PVC), 30–70% wood particles (softwood/hardwood flour or shavings), and 0.5–15% additives, and are designed for long-term performance, shape flexibility, good stiffness and working properties [74,75]. Classic, fossil-based plastics are biologically inert materials, but this property is only partially transferred to WPC because the plastic matrix cannot totally encapsulate the wood particles, which can absorb moisture to levels that make them susceptible to fungal attack [70]. While microbiological attack is less of a problem for most indoor applications, it is of great importance for WPC durability in outdoor applications. Fungi, especially white rots, can rapidly colonize and decay WPC under favorable conditions [76]. Candelier et al. [77] reported that a WPC composed of BIOPLAST GS2189 biopolymer (from PLA and potato starch) and spruce sawdust provided good resistance to fungal and termite attack, but resistance decreased slightly as wood content increased to 30%, while the mass loss for pure polymer was 0% according to the European standard EN117. The biodegradation of low-density polyethylene (LDPE)/alkali-treated corn flour composites exposed to soil burial for 6 months also increased with biomass component level [78]. In both cases, increased biomass reduces the potential for the polymer to completely encapsulate the biomass to reduce moisture uptake and microbial access.

**Figure 4:**
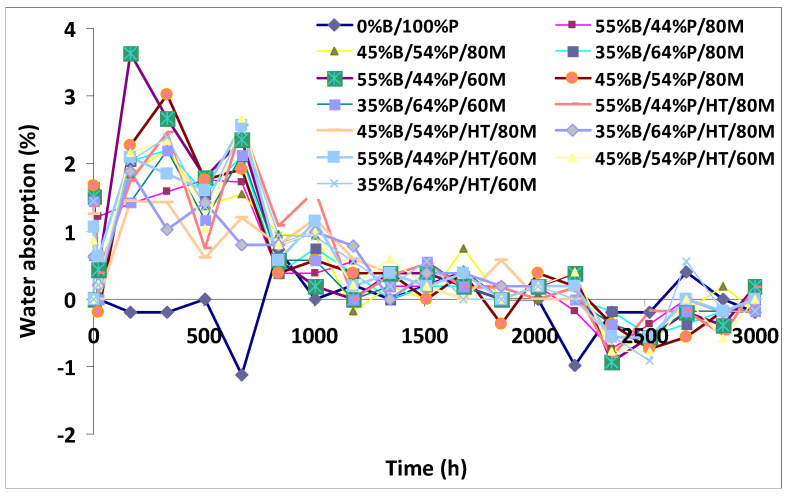
Effect of bamboo content on water absorption of bamboo (B)/plastic (P) composites composed of different plastics. M: mesh size, HT: heat treatment.

Dehghan [79,80,81] investigated the biological decay resistance of different bamboo/PLA composites (BPC’s) and found that moisture contents increased to approximately 3 % within 28 days (672 h) (Figure 4) and that the weight losses were elevated at all ratios tested (Table 6).

Schirp and Wolcott [82] found that mass loss was a more sensitive indicator of fungal decay than strength and stiffness measurements, possibly because the high-density polyethylene (HDPE) was more dominant in the flexural properties while it was unaffected by fungal attack. Ascomycete attack of LDPE composites containing 30% of various biomass fillers was strongly influenced by the aspect and composition of fillers. Particles with higher length-to-diameter ratios experienced more mold growth as did materials with fillers containing soluble or easy hydrolysable fractions (milled straw of seed flax and hydrolyzed keratin of bird feathers) that provided easily available carbon sources for microbial growth [82]. The polymer content also affects degradability. Polymer composites with 50% hazelnut husk flour were more resistant to decay with the addition of HDPE. However, the incorporation of polypropylene or polyethylene in combination with maleic anhydride resulted in more susceptible composites [83].

While of natural origin, PLA is hydrophobic like the fossil-based equivalent polyolefins. It has poor surface adhesion within more polar natural fibers or fillers and requires compatibilizing agents to stabilize the interface [84,85]. Chitosan, especially in high amounts, increases the hydrophilic nature of PLA composites, favoring moisture uptake and microbial attack leading to relatively small mass losses, but significant embrittlement and changes in mechanical, thermal, and surface properties were observed [86].

## 7. Conclusions

Biocomposites are increasingly used to produce machinery, automotive components, packaging, and construction products. While these materials are more environmentally friendly than similar petrochemical-derived products, they still have some practical limitations, including reduced mechanical strength, lower physical properties, and susceptibility to biological degradation. However, the continued development of more sustainable bio hybrid composites will help to improve the properties of these hybrid materials. This approach will result in more environmentally friendly production, use, and disposal that is more compatible with use in the circular economy. Moreover, the development of novel biocomposites with controlled decomposition at the end of their life cycle will minimize uncontrolled waste accumulation in landfill.

## Figures and Tables

**Table 1:** Fundamental properties of bioplastics derived from different organisms that are used for biocomposites.

Organisms	Compound	Abbreviation	Properties
All plant cells	Cellulose	-	High thermal stability, high ductility, optical transparency [4]
All plant cells	Lignin	-	High mechanical properties and high rigidity [5]
All higher fungi, arthropods, molluscs, cephalopod beaks, scales of fish and lissamphibians	Chitin	-	Translucent, resilient high ductility [4]
*Pseudomonas oleovorans, P. putida*	Poly(3-hydroxyalkanoate)	PHA	High ductility but brittle [5]
*Bacillus megaterium, Ralstonia* *eutropha*	Poly(3-hydroxybutyrate)	PHB	Non-transparent, outstanding ductility and toughness, completely biodegradable, moisture-sensitive [5]
*Laetiporus sulphureus, Laccaria bicolor, Phanerochaete chrysosporium, Bacillus* spp., *Lactobacillus* spp.	Polylactic acid	PLA	Transparent, water-repellent, printable, and breathable [6]
Maize, wheat, potatoes, tapioca	Thermoplastic starch	TPS	Soluble in water, poor thermal resistance, low strength [7]

**Table 2:** Chemical composition of some common fibers (% of total) [12].

Type of Fiber	Cellulose	Lignin	Pentosan	Ash	Silica
Stalk fiber	28–48				
Rice	29–51	12–16	23–28	15–20	9–14
Wheat	31–45	16–21	26–32	4.5–9	3–7
Barley	31–48	14–15	24–29	5–7	3–6
Qat	33–50	16–19	27–38	6–8	4–6.5
Rye		16–19	27–30	2–5	0.5–4
Cane fiber	32–48				
Bagasse	26–43	19–24	27–32	1.5–5	0.7–3.5
Bamboo		21–31	15–26	1.7–5	0.7
Grass fiber	33–38				
Esparto	-	17–19	27–32	6–8	-
Sabai		22	24	6	-
Red fiber	44–46				
*Phragmites communis*		22–24	20	3	2
Bast fiber	43–47				
Seed flax	44–57	21–23	24–26	5	-
Kenaf	45–63	15–19	22–23	2–5	-
Jute	57–77	21.26	18.21	0.5–2	-
Hemp	87–91	9–13	14–17	0.8	-
Ramie		-	5–8	-	-
Core fiber	37–49				
Kenaf	41–48	15–21	18–24	2–4	-
Jute		21–24	18–22	0.8	-
Leaf fiber	56–63				
Abaca(Manila)	47–62	7–9	15–17	3	-
Sisal (agave)		7–9	21–24	0.6–1	-
Seed hull fiber	85–90				
Cotton		0.7–1.6	1–3	0.8–2	-
Wood fiber	40–45				
Coniferous	48–49	26–34	7–14	<1	-
Deciduous		23–30	16–26	<1	-

**Table 3:** Examples of potential hybrid and reinforcement materials for common composites as well as biocomposites [30].

Matrix	Reinforcement	Biocomposites	Examples
Polylactic acid	Biofibers (Hemp, Sisal, Jute, etc.)	[Biodegradable] Biofiber-reinforced bio-based-polymer	Sisal/PLA, Biofiber/Starch, Hemp/PBS
Poly(3-hydroxybutyrate-co-3-hydroxyvalerate)	Nanocellulose (Plant or Bacteria)		
Poly(butylene succinate)(PBS)	Chitosan		Kenaf/PP, Jute/PE
Thermoplastic starch	Wool/Silk	[Non-Biodegradable] Biofiber-reinforced petro-based polymers Synthetic fibers (glass or carbon fibers)-reinforced bio-based-polymers	
Poly(butylene adipate-co-terephthalate)	Biosourced Carbon		CF/PBS, GF/PLA
Polypropylene	Industry Co-product		
Polyethylene	Synthetic fibers (eg. Glass fiber (GF), Carbon fiber (CF))		

**Table 4:** Mechanical properties of natural fibers [33].

Fiber	Tensile Strength (MPa)	Youngs Modulus (GPa)	Elongation at Break (%)
OPEFB *	248	3.2	2.5
Flax	88–1500	60–80	1.2–1.6
Hemp	550–900	70	1.6
Jute	400–800	10–30	1.8
Ramie	500	44	2
Coir	220	6	15–25
Sisal	600–700	38	2–3
Abaca	980	---	---
Cotton	400	12	3–10
Kenaf (bast)	295	---	2.7–6.9
Kenaf (core)	---	---	---
Bagasse	20–290	19.7–27.1	1.1
Henequen	430–580	---	3–4.7
Pineapple	170–1672	82	1–3
Banana	355	33.8	5.3

* Oil Palm Empty Fruit Bunch.

**Table 5:** Properties of representative biocomposites and their hybrids with or without maleic anhydride–grafted polyethylene (MAPE) or polypropylene (MAPP) [47].

Resin	Filler	Impact Strength (kJ/m^2^)	Tensile Strength(MPa)	Tensile Modulus(GPa)	Comments	Reference
Plastic waste (PE and PP)	Wood flour	2.9–6.2 Unnotch	6–13	2.3–3.9	MAPE compatibilization and lubricant utilization	[48]
PP	Wood, poultry litter biochar	8.1 Notch	27	4.3	Hybrid biocomposites–MAPP compatibilization	[49]
PP	Flax fiber	751 Unnotch	40	6.5	Needle-punch fiber mat composite	[50]
Waxy maize starch	Neat and modified liquid crystalline cellulose, microcrystalline cellulose	-	505–790	22–32	Starch/cellulose hybrid biocomposites	[51]
Epoxy/acrylate	Glass fiber	237 Notch	532	37	Methacrylated epoxidized sucrose soyate resin/glass fiber	[52]
Bio-polyurethane (Bio-PU)	Sisal fiber	-	57–119	1.2–2.2	Rubber seed oil polyurethane	[53]
PBS/PLA	Flax fiber	9.1–17.8 Notch	39–55	3.6–7.4	Fully biodegradable composite	[54]
PLA	Carbon fibers, twisted yarns of jute fibers	-	57–185	5.1–19.5	Continuous fiber reinforcement probed by 3D printing	[55]

**Table 6:** Biological decomposition of bamboo/plastic composites composed of different plastics [79].

Treatment	Treatment and Mesh Size	Fungi (Mass Losses %)		
		Gloeophyllum Trabeum	Trametes Versicolor	Chaetomium Globosum
A	0%B/100%P	1.34 ± 0.39	0.60 ± 0.05	0.75 ± 0.63
B	55%B/44%P/80M	24.62 ± 0.80	3.65 ± 0.96	3.29 ± 0.65
C	45%B/54%P/80M	25.38 ± 5.49	2.65 ± 1.69	2.97 ± 0.74
D	35%B/64%P/80M	22.80 ± 3.98	2.27 ± 0.58	10.81 ± 6.49
E	55%B/44%P/60M	26.86 ± 2.70	2.64 ± 0.47	2.60 ± 2.34
F	45%B/54%P/80M	18.28 ± 2.26	2.28 ± 0.11	4.12 ± 3.79
G	35%B/64%P/60M	21.97 ± 0.81	3.57 ± 0.90	3.42 ± 1.79
H	55%B/44%P/HT/80M	24.24 ± 1.48	2.00 ± 0.19	2.37 ± 1.75
I	45%B/54%P/HT/80M	23.56 ± 1.96	2.20 ± 0.70	4.98 ± 2.50
J	35%B/64%P/HT/80M	20.12 ± 1.67	2.98 ± 0.41	5.77 ± 4.38
K	55%B/44%P/HT/60M	19.77 ± 2.29	2.27 ± 0.58	2.64 ± 1.14
L	45%B/54%P/HT/60M	22.91 ± 0.33	2.44 ± 1.01	3.25 ± 2.69
M	35%B/64%P/HT/60M	21.98 ± 3.25	3.06 ± 0.90	2.08 ± 1.33

B: bamboo; P: plastic; M: mesh size, HT: heat treatment; ± Values represent standard deviations of the means.

## Data Availability

All data reviewed or analyzed during this study are included in this published article.

## References

[1] Geyer R., Jambeck J.R., Law K.L. (2017). Production, use, and fate of all plastics ever made. Sci. Adv..

[2] Komaragounder S., Santhoskumar A.U., Palanivelu K., Sharma S.K., Nayak S.K. (2010). Comparison of Biological Activity Transistion Metal 12 Hydroxy oleate on Photodegradation of Plastics. J. Bioremediation Biodegrad..

[3] Umapathi A. (2010). A New Synthesis of Nickel 12-Hydroxy Oleate Formulation to Improve Polyolefin’s Degradation. J. Bioremediation Biodegrad..

[4] Brigham C. (2018). Biopolymers: Biodegradable alternatives to traditional plastics. Green Chemistry.

[5] Yu J. (2007). Microbial production of bioplastics from renewable resources. Bioprocessing for Value-Added Products from Renewable Resources.

[6] Varsha Y.S., Varsha Y. (2011). Overview on Polyhydroxyalkanoates: A Promising Biopol. J. Microb. Biochem. Technol..

[7] Cyras V.P., Manfredi L.B., Ton-That M.T., Vazquez A. (2008). Physical and mechanical properties of thermoplastic starch/montmorillonite nanocomposite films. Carbohyd. Polym..

[8] Kumar M., Singhal A., Verma P.K., Thakur I.S. (2017). Production and Characterization of Polyhydroxyalkanoate from Lignin Derivatives by Pandoraea sp. ISTKB. ACS Omega.

[9] Avérous L., Pollet E., Avérous L., Pollet E. (2012). Green Nano-Biocomposites. Environmental Silicate Nano-Biocomposites.

[10] Vieira M.G.A., da Silva M.A., Santos L., Beppu M.M. (2011). Natural-based plasticizers and biopolymer films: A review. Eur. Polym. J..

[11] Patel J.P., Parsania P.H., Navinchandra G.S. (2018). Characterization, testing, and reinforcing materials of biodegradable composites. Biodegradable and Biocompatible Polymer Composites.

[12] Han J.S., Rowell J.S., Rowell R.A., Young J.K., Rowell (1996). Chemical composition of fibers. Paper and Composites from Agro-Based Resources.

[13] Lau A.K.-T., Cheung K.H.Y., Lau A.K., Hung A.P. (2017). Natural fiber-reinforced polymer-based composites. Natural Fiber-Reinforced Biodegradable and Bioresorbable Polymer Composites.

[14] Mihai M., Ton-That M.-T. (2013). Novel polylactide/triticale straw biocomposites: Processing, formulation, and properties. Polym. Eng. Sci..

[15] Aslan M., Tufan M., Küçükömeroğlu T. (2018). Tribological and mechanical performance of sisal-filled waste carbon and glass fibre hybrid composites. Compos. Part B Eng..

[16] Réquilé S., Le Duigou A., Bourmaud A., Baley C. (2018). Peeling experiments for hemp retting characterization targeting biocomposites. Ind. Crop. Prod..

[17] Martin N., Mouret N., Davies P., Baley C. (2013). Influence of the degree of retting of flax fibers on the tensile properties of single fibers and short fiber/polypropylene composites. Ind. Crop. Prod..

[18] Väisänen T., Batello P., Lappalainen R., Tomppo L. (2018). Modification of hemp fibers (*Cannabis sativa* L.) for composite applications. Ind. Crop. Prod..

[19] Hamidon M.H., Sultan M.T., Ariffin A.H., Shah A.U. (2019). Effects of fibre treatment on mechanical properties of kenaf fibre reinforced composites: A review. J. Mater. Res. Technol..

[20] Atiqah A., Maleque M., Jawaid M., Iqbal M. (2013). Development of kenaf-glass reinforced unsaturated polyester hybrid composite for structural applications. Compos. Part B Eng..

[21] Kipriotis E., Heping X., Vafeiadakis T., Kiprioti M., Alexopoulou E. (2015). Ramie and kenaf as feed crops. Ind. Crop. Prod..

[22] Chandra R., Rustgi R. (1998). Biodegradable polymers. Prog. Polym. Sci..

[23] Quitadamo A., Massardier V., Valente M. (2019). Eco-Friendly Approach and Potential Biodegradable Polymer Matrix for WPC Composite Materials in Outdoor Application. Int. J. Polym. Sci..

[24] Fowler P.A., Hughes J.M., Elias R.M. (2006). Biocomposites: Technology, environmental, credentials and market forces. J. Sci. Food Agric..

[25] Bhatia J.K., Kaith B.S., Kalia S., Kalia S. (2016). Recent Developments in Surface Modification of Natural Fibers for Their Use in Biocomposites. Biodegradable Green Composites.

[26] Akampumuza O., Wambua P.M., Ahmed A., Li W., Qin X.-H. (2016). Review of the applications of biocomposites in the automotive industry. Polym. Compos..

[27] Ahmad F., Choi H.S., Park M.K. (2014). A Review: Natural Fiber Composites Selection in View of Mechanical, Light Weight, and Economic Properties. Macromol. Mater. Eng..

[28] Shalwan A., Yousif B. (2012). In State of Art: Mechanical and tribological behaviour of polymeric composites based on natural fibres. Mater. Des..

[29] Sassoni E., Manzi S., Motori A., Montecchi M., Canti M. (2014). Novel sustainable hemp-based composites for application in the building industry: Physical, thermal and mechanical characterization. Energy Build..

[30] Chang B.P., Mohanty A.K., Misra M. (2020). Studies on durability of sustainable biobased composites: A review. RSC Adv..

[31] Tserki V., Matzinos P., Zafeiropoulos N.E., Panayiotou C. (2006). Development of biodegradable composites with treated and compatibilized lignocellulosic fibers. J. Appl. Polym. Sci..

[32] Koncar V. (2019). Smart Textiles for In Situ Monitoring of Composites.

[33] Jawaid M., Khalil H.A. (2011). Cellulosic/synthetic fibre reinforced polymer hybrid composites: A review. Carbohydr. Polym..

[34] Baghaei B., Skrifvars M.O.V. (2016). Characterisation of polylactic acid biocomposites made from prepregs composed of woven polylactic acid/hemp–Lyocell hybrid yarn fabrics. Compos. Part A Appl. Sci. Manuf..

[35] Satyanarayana K.G., Arizaga G.G.C., Wypych F. (2009). Biodegradable composites based on lignocellulosic fibers—An overview. Prog. Polym. Sci..

[36] O’Donnell A., Dweib M., Wool R. (2004). Natural fiber composites with plant oil-based resin. Compos. Sci. Technol..

[37] Dweib M., Hu B., O’Donnell A., Shenton H., Wool R. (2004). All natural composite sandwich beams for structural applications. Compos. Struct..

[38] Hashemi S. (2008). Hybridisation effect on flexural properties of single- and double-gated injection moulded acrylonitrile butadiene styrene (ABS) filled with short glass fibres and glass beads particles. J. Mater. Sci..

[39] Bansal G., Singh V.K., Patil P., Rastogi S. (2016). Water absorption and thickness swelling characterization of chicken feather fiber and extracted fish residue powder filled epoxy based hybrid biocomposite. Int. J. Waste Resour..

[40] Júnior C.P., de Carvalho L., Fonseca V., Monteiro S., D’Almeida J. (2004). Analysis of the tensile strength of polyester/hybrid ramie–cotton fabric composites. Polym. Test..

[41] Sanjay M.R., Arpitha G.R., Naik L.L., Gopalakrishna K., Yogesha B. (2016). Applications of Natural Fibers and Its Composites: An Overview. Nat. Resour..

[42] Madhu P., Sanjay M.R., Senthamaraikannan P., Pradeep S., Siengchin S., Jawaid M., Kathiresan M. (2018). Effect of Various Chemical Treatments of Prosopis juliflora Fibers as Composite Reinforcement: Physicochemical, Thermal, Mechanical, and Morphological Properties. J. Nat. Fibers.

[43] Retuert J., Quijada R., Arias V., Yazdani-Pedram M. (2003). Porous silica derived from chitosan-containing hybrid composites. J. Mater. Res..

[44] Ahmed K.S., Vijayarangan S. (2008). Tensile, flexural and interlaminar shear properties of woven jute and jute-glass fabric reinforced polyester composites. J. Mater. Process. Technol..

[45] Gujjala R., Ojha S., Acharya D.K., Pal S. (2013). Mechanical properties of woven jute–glass hybrid-reinforced epoxy composite. J. Compos. Mater..

[46] Ramesh M., Palanikumar K., Reddy K.H. (2013). Mechanical property evaluation of sisal–jute–glass fiber reinforced polyester composites. Compos. Part B Eng..

[47] Mohanty A.K., Vivekanandhan S., Pin J.-M., Misra M. (2018). Composites from renewable and sustainable resources: Challenges and innovations. Science.

[48] Turku I., Keskisaari A., Kärki T., Puurtinen A., Marttila P. (2017). Characterization of wood plastic composites manufactured from recycled plastic blends. Compos. Struct..

[49] Das O., Sarmah A.K., Bhattacharyya D. (2016). Biocomposites from waste derived biochars: Mechanical, thermal, chemical, and morphological properties. Waste Manag..

[50] Oksman K. (2000). Mechanical Properties of Natural Fibre Mat Reinforced Thermoplastic. Appl. Compos. Mater..

[51] Rahman M.M., Netravali A.N. (2018). Advanced Green composites using liquid crystalline cellulose fibers and waxy maize starch based resin. Compos. Sci. Technol..

[52] Hosseini N., Webster D.C., Ulven C. (2016). Advanced biocomposite from highly functional methacrylated epoxidized sucrose soyate (MAESS) resin derived from vegetable oil and fiberglass fabric for composite applications. Eur. Polym. J..

[53] Bakare I., Okieimen F., Pavithran C., Khalil H.A., Brahmakumar M. (2010). Mechanical and thermal properties of sisal fiber-reinforced rubber seed oil-based polyurethane composites. Mater. Des..

[54] Bourmaud A., Corre Y.-M., Baley C. (2015). Fully biodegradable composites: Use of poly-(butylene-succinate) as a matrix and to plasticize l-poly-(lactide)-flax blends. Ind. Crop. Prod..

[55] Matsuzaki R., Ueda M., Namiki M., Jeong T.-K., Asahara H., Horiguchi K., Nakamura T., Todoroki A., Hirano Y. (2016). Three-dimensional printing of continuous-fiber composites by in-nozzle impregnation. Sci. Rep..

[56] Alvarez-Chavez C.R., Edwards S., Moure-Eraso R., Geiser K. (2012). Sustainability of bio-based plastics: General comparative analysis and recommendations for improvement. J. Clean. Prod..

[57] AL-Oqla F.M., Omari M.A., Jawaid M., Sapuan S.M., Alothman O.Y. (2017). Green Biocomposites. Manufacturing and Properties.

[58] Steinmetz Z., Wollmann C., Schaefer M., Buchmann C., David J., Troger J., Munoz K., Fror O., Schaumann G.E. (2016). Plastic mulching in agriculture. Trading short-term agronomic benefits for long-term soil degradation?. Sci. Total Environ..

[59] Bari E., Morrell J.J., Sistani A., Jawaid M., Thariq M., Saba N. (2019). Durability of natural/synthetic/biomass fiberebased polymeric composites: Laboratory and field tests. Durability and Life Prediction in Biocomposites, Fibre—Reinforced Composites and Hybrid Composites, Woodhead Publishing Series in Composites Science and Engineering.

[60] Beg M.D.H., Pickering K. (2008). Accelerated weathering of unbleached and bleached Kraft wood fibre reinforced polypropylene composites. Polym. Degrad. Stab..

[61] Das R., Karumbaiah K.M., Fakirov S. (2015). Biodegradable polyester-based blends and composites: Manufacturing, properties and applications. Biodegradable Polyesters.

[62] Hidayat A., Tachibana S. (2012). Characterization of polylactic acid (PLA)/kenaf composite degradation by immobilized mycelia of Pleurotus ostreatus. Int. Biodeterior. Biodegrad..

[63] Zhao Y.-Q., Cheung H.-Y., Lau K.-T., Xu C.-L., Zhao D.-D., Li H.-L. (2010). Silkworm silk/poly(lactic acid) biocomposites: Dynamic mechanical, thermal and biodegradable properties. Polym. Degrad. Stab..

[64] European Standard (2000). 13432:2000 Packaging—Requirements for Packaging Recoverable through Composting and Biodegradation—Test Scheme and Evaluation Criteria for the Final Acceptance of Packaging.

[65] Gurunathan T., Mohanty S., Nayak K. (2015). A review of the recent developments in biocopmosites based on natural fibres and their application perspectives. Compos. Part A.

[66] Lu H., Madbouly S.A., Schrader J.A., Kessler M.R., Grewell D., Graves W.R. (2014). Novel bio-based composites of polyhydroxyalkanoate (PHA)/distillers dried grains with solubles (DDGS). RSC Adv..

[67] Cunha M., Berthet M.-A., Pereira R.N., Covas J.A., Vicente A., Hilliou L. (2014). Development of polyhydroxyalkanoate/beer spent grain fibers composites for film blowing applications. Polym. Compos..

[68] Byrom D. (1993). The synthesis and biodegradation of polyhydroxyalkanoates from bacteria. Int. Biodeterior. Biodegrad..

[69] Muniyasamy S., John M.J., Thakur V.K., Thakur M.K., Kessler M.R. (2017). Biodegradability of Biobased Polymeric Materials in Natural Environments. Handbook of Composites from Renewable Materials.

[70] Shah A.A., Hasan F., Hameed A., Ahmed S. (2008). Biological degradation of plastics: A comprehensive review. Biotechnol. Adv..

[71] Rutkowska M., Heimowska A., Krasowska K., Janik H. (2002). Biodegradability of Polyethylene Starch Blends in Sea Water. Pol. J. Environ. Stud..

[72] Ruka D.R., Sangwan P., Garvey C.J., Simon G.P., Dean K.M. (2015). Biodegradability of poly-3-hydroxybutyrate/bacterial cellulose composites under aerobic conditions—Measured via evolution of carbon dioxide, spectroscopic and diraction methods. Environ. Sci. Technol..

[73] Brebu M. (2020). Environmental Degradation of Plastic Composites with Natural Fillers—A Review. Polymers.

[74] Taylor A., Yadama V., Englund K.R., Harper D. (2009). Wood Plastic Composites—A Primer.

[75] Chan C.M., Vandi L.-J., Pratt S., Halley P., Richardson D., Werker A., Laycock B. (2017). Composites of Wood and Biodegradable Thermoplastics: A Review. Polym. Rev..

[76] Schirp A., Wolcott M.P. (2005). Influence of fungal decay and moisture absorption on mechanical properties of extruded wood-plastic composites. Wood Fiber Sci..

[77] Candelier K., Atli A., Alteyrac J. (2018). Termite and decay resistance of bioplast-spruce green wood-plastic composites. Eur. J. Wood Wood Prod..

[78] Sahi S., Djidjelli H., Boukerrou A. (2016). Biodegradation study of bio-corn flour filled low density polyethylene composites assessed by natural soil. J. Polym. Eng..

[79] Dehghan M. (2018). Physical, Mechanical and Biological Resistance of Bamboo Flour-Polylactic Acid (PLA) Biocomposite. Master’s Thesis.

[80] Dehghan M., Faezipour M., Azizi M., Hosseinabadi H.Z., Bari E., Nicholas D.D. (2019). Assessment of physical, mechanical, and biological properties of bamboo plastic composite made with polylactic acid. Maderas Cienc. Tecnol..

[81] Dehghan M., Faezipour M., Azizi M., Bari E. (2019). Assesement of the Biodegradability of Composites produced from Poly-Lactic Acid and Bamboo Flour. Iran. J. Wood Pap. Ind..

[82] Mastalygina E.E., Popov A.A., Pantyukhov P.V. (2017). Effect of biobased fillers nature on biodeterioration of hybrid polyethylene composites by mould fungi. IOP Conf. Ser. Mater. Sci. Eng..

[83] Tufan M., Akbas S., Guleç T., Tasçıoglu C., Alma M.H. (2015). Mechanical, thermal, morphological properties and decay resistance of filled hazelnut husk polymer composites. Maderas Cienc. Tecnol..

[84] Zhang J.-F., Sun X. (2004). Mechanical properties of poly(lactic acid)/starch composites compatibilised by maleicanhydride. Biomacromolecules.

[85] Vaidya U.R., Battacharya M. (1994). Method of grafting functional groups to synthetic polymers for making biodegradable plastics. J. Appl. Polym. Sci..

[86] Vasile C., Pamfil D., Râpa M., Darie-Ni¸ta R.N., Mitelut A.C., Popa E.E., Popescu P.A., Draghici M.C., Popa M.E. (2018). Study of the soil burial degradation of some PLA/CS Biocomposites. Compos. Part B.

